# Liproxstatin-1 Protects Hair Cell-Like HEI-OC1 Cells and Cochlear Hair Cells against Neomycin Ototoxicity

**DOI:** 10.1155/2020/1782659

**Published:** 2020-12-01

**Authors:** Zhiwei Zheng, Dongmei Tang, Liping Zhao, Wen Li, Jinghong Han, Bing Hu, Guohui Nie, Yingzi He

**Affiliations:** ^1^ENT Institute and Department of Otorhinolaryngology, Eye & ENT Hospital, Fudan University, Shanghai 200031, China; ^2^NHC Key Laboratory of Hearing Medicine (Fudan University), Shanghai 200031, China; ^3^Department of Otolaryngology and Institute of Translational Medicine, Shenzhen Second People's Hospital/The First Affiliated Hospital of Shenzhen University Health Science Center, Shenzhen 518035, China

## Abstract

Ferroptosis is a recently discovered iron-dependent form of oxidative programmed cell death distinct from caspase-dependent apoptosis. In this study, we investigated the effect of ferroptosis in neomycin-induced hair cell loss by using selective ferroptosis inhibitor liproxstatin-1 (Lip-1). Cell viability was identified by CCK8 assay. The levels of reactive oxygen species (ROS) were determined by DCFH-DA and cellROX green staining. The mitochondrial membrane potential (ΔΨ*m*) was evaluated by TMRM staining. Intracellular iron and lipid peroxides were detected with Mito-FerroGreen and Liperfluo probes. We found that ferroptosis can be induced in both HEI-OC1 cells and neonatal mouse cochlear explants, as evidenced by Mito-FerroGreen and Liperfluo staining. Further experiments showed that pretreatment with Lip-1 significantly alleviated neomycin-induced increased ROS generation and disruption in ΔΨ*m* in the HEI-OC1 cells. In parallel, Lip-1 significantly attenuated neomycin-induced hair cell damage in neonatal mouse cochlear explants. Collectively, these results suggest a novel mechanism for neomycin-induced ototoxicity and suggest that ferroptosis inhibition may be a new clinical intervention to prevent hearing loss.

## 1. Introduction

Hearing loss can be caused by ototoxic pharmaceutical agents, excessive noise, genetic disorders, and aging. Aminoglycoside antibiotics are one of the largest classes of valuable clinical agents with ototoxic adverse effects [1, 2]. Nonmammalian vertebrates can regenerate hair cells, such that ototoxic damage is not permanent in these taxa, while ototoxic drugs can result in irreversible damage to the hair cells within the mammalian inner ear leading to hearing loss. The crucial mechanism responsible for aminoglycoside-induced ototoxicity is oxidative stress [3]. Overproduction of reactive oxygen species (ROS) resulting from oxidative stress overwhelms the ROS defense and disturbs the redox balance, triggering mitochondrial depolarization, activating caspase-3, and eventually inducing hair cell injury [1, 4]. However, this mechanism is not exclusively responsible for aminoglycoside-induced hair cell death [5–7]. Thus, better understanding of the mechanisms of aminoglycoside-induced ototoxicity is crucial for developing a new promising treatment strategy to prevent hearing loss.

Ferroptosis is a recently discovered novel type of iron-dependent programmed cell death that is characterized by the intracellular overproduction of ROS and lipid peroxidation, but independent from caspase-mediated cell death, autophagy, and necrosis [8, 9]. Multiple inducers, regulators, and inhibitors of ferroptosis have been shown to regulate the accumulation of ROS in an iron-dependent manner [10]. Known inducers of ferroptosis can be divided into two classes: class 1 ferroptosis inducers, including erastin, sulfasalazine, and sorafenib, which can trigger ferroptotic cell death by inhibiting the activity of system X_c_^−^, the glutamate/cystine antiporter, which leads to the depletion of intracellular glutathione (GSH), a major cellular antioxidant, and results in inactivation of glutathione peroxidase-4 (GPX4), a lipid hydroperoxide detoxifying enzyme required for the clearance of endogenous lipid ROS [8, 10, 11]; class 2 ferroptosis inducers, including Ras-selective lethal 3 (RSL3) and FIN56, which can directly inhibit GPX4 without depleting GSH [8, 10, 11]. Loss of GPX4 activity induces lipid ROS overaccumulation and eventually induces cell death [10, 11]. Additionally, several small-molecule compounds have been identified as inhibitors of ferroptosis, including Lip-1, a ferroptosis inhibitor and a lipid ROS scavenger, deferoxamine (DFO), an iron chelator, and FINO_2_, an oxidized iron inhibitor [8]. Ferroptosis has been identified in various pathological processes, such as ischemia-reperfusion (I/R) injury [12], acute kidney injury [13–15], neurotoxicity [13], and cancer [12]. Furthermore, a recent report [12] suggested that ferroptosis is associated with the pathogenesis of I/R injury and that inhibitors of glutaminolysis protect the heart against ischemia-reperfusion-induced injury and are thus a potential therapeutic target. However, the specific molecular mechanisms underlying the induction of ferroptosis in hair cell survival remain unknown. This study's aim was to investigate if ferroptosis is associated with aminoglycoside-induced ototoxicity in HEI-OC1 cell line and in an *in vitro* neonatal mouse cochlear model.

## 2. Materials and Methods

### 2.1. HEI-OC1 Cell Culture

The House Ear Institute-Organ of Corti 1 (HEI-OC1) cell line is a widely used auditory HC line [16–20]. Cells were cultured in high-glucose DMEM (Gibco BRL, Gaithersburg, MD, USA) supplemented with 5% FBS (Gibco BRL) in acceptable conditions (33°C, 5% CO_2_).

### 2.2. Postnatal Cochlear Explants

All animal experiments were approved by the Shanghai Medical Experimental Animal Administrative Committee. Cochleae from C57BL/6 mice at postnatal day (P) 2 were dissected in phosphate-buffered saline (PBS). The cochlear explants were stuck to a glass coverslip coated with Cell-Tak (BD Biosciences, Franklin Lakes, NJ, USA). Cochlear explants were incubated in DMEM/F12 medium supplemented with N2/B27 (Invitrogen) and ampicillin at 37°C in a 5% CO_2_/95% air atmosphere overnight prior to each treatment.

### 2.3. Drug Treatments

RSL3, Lip-1, N-acetylcysteine amide, and z-VAD-FMK were purchased from Selleck Chemicals (Houston, TX) and were initially dissolved in DMSO and diluted in the culture medium (DMEM supplemented with 5% FBS) to a final concentration. Neomycin was purchased from Sigma-Aldrich (Saint Louis, USA).

### 2.4. Cell Viability

Cell Counting Kit-8 (CCK8) was used to examine cell viability according to the manufacturer's instructions. In brief, HEI-OC1 cells were seeded at a density of 5000 cells/well in 96-well plates in three replicates and incubated overnight. After treatment, CCK8 (Sigma, Saint Louis, USA) was added to each well for 4 h. The optical density (OD) values were measured at 450 nm using a plate reader (Bio-Rad).

### 2.5. Annexin V-FITC/PI Assay

Annexin V-FITC and propidium iodide (PI) assay was detected by flow cytometry using Annexin V-FITC/PI (BD Biosciences, Piscataway, NJ, USA) according to the manufacturer's instructions. Cells were collected by centrifugation at 3000 × g for 5 min and resuspended gently in 1x binding buffer at a concentration of 1 × 10^6^ cells/ml. Annexin V-FITC (5 *μ*l) and PI (5 *μ*l) were gently mixed with cell suspension and incubated for 15 min at room temperature in the dark. The cells were immediately analyzed by flow cytometry.

### 2.6. ROS Assay

ROS production was detected using cellROX green reagent (Molecular Probes, Life Technologies, USA) and DCFH-DA (Molecular Probes, USA). We followed the methods of He et al. [21]. Briefly, after treatment, cells were washed with prewarmed PBS and stained with 5 *μ*M cellROX green or 50 *μ*M DCFH-DA for 30 min or 10 min, respectively, and Hoechst (1 *μ*g/ml) for 15 min at 37°C. Fluorescence was then observed using a fluorescence microscope. All images are representative of three independent experiments.

### 2.7. Mitochondrial Transmembrane Potential Measurement

Mitochondrial membrane potential (ΔΨ*m*) was estimated by TMRM (Molecular Probes, Invitrogen, UK) staining. Samples were treated with the designated conditions and then incubated with 20 nM TMRM for 30 min at 37°C.

### 2.8. Mitochondrial Morphology Assay

Mitochondrial morphology was measured with 20 nM MitoTracker Green FM probe (Molecular Probes, Invitrogen, UK), according to the manufacturer's instructions. Briefly, cells were stained with MitoTracker Green for 30 min and Hoechst (1 *μ*g/ml) for 15 min at 37°C.

### 2.9. Iron Staining

To label iron in mitochondria, samples were incubated with Mito-FerroGreen (Dojindo, Kumamoto, Japan) in accordance with the manufacturer's instructions. Briefly, samples were washed twice with serum-free DMEM. Mito-FerroGreen was freshly dissolved in DMSO and added at a final concentration of 5 *μ*M. Samples were incubated with the reagent for 30 min at 37°C.

### 2.10. Lipid Peroxide Measurement

To detect lipid peroxides, samples were exposed for 30 min at 37°C to Liperfluo (Dojindo, Kumamoto, Japan) at a final concentration of 5 *μ*M in accordance with the manufacturer's instructions.

### 2.11. TUNEL Assay

TUNEL assay was determined by *in situ* cell detection kit (Roche, Nutley, NJ, USA) according to the manufacturer's instructions. Briefly, cells were stained with TUNEL reaction mixture at 37°C for 30 min in a humid atmosphere.

### 2.12. Immunofluorescence

Cochleae fixed in 4% paraformaldehyde (PFA) were rinsed three times with PBS and permeabilized with 1% Triton X-100 in PBS (PBST) for 30 min at room temperature. Permeabilized explants were then blocked with 10% donkey serum in PBST for 1 h followed by incubation with the anti-myosin 7a antibody (Proteus Biosciences, Ramona, CA, USA), anti-cleaved caspase-3 antibody (Cell Signaling Technology, Inc., Danvers, MA, USA), and anti-parvalbumin antibody (Abcam, Cambridge, MA, USA) overnight at 4°C. The explants were then washed three times with PBS and incubated with secondary fluorescent antibodies for 1 h at 37°C in the dark. Nuclei were labeled with DAPI for 10 min at room temperature, and the samples were visualized under a Leica SP8 confocal fluorescence microscope (Leica Microsystems, Biberach, Germany).

### 2.13. Western Blot Analysis

Cochleae were lysed with cold RIPA lysis buffer plus PMSF. Equal amounts of each protein sample were separated via 12% SDS-PAGE and transferred to PVDF membranes (Immobilon-P, Millipore, Schaffhausen, Switzerland). After blocking with 5% skim milk for 1 h, the membranes were probed with anti-GPX4 (1 : 1000, Abcam, Cambridge, MA, USA) and anti-GAPDH (1 : 4000) overnight at 4°C. Protein bands were detected using a chemiluminescence solution, ECL kit (Millipore, USA). Each experiment was repeated three times, and all protein expression was normalized to that of GAPDH.

### 2.14. Cell Counts

Hair cells labeled with myosin 7a were considered to be surviving hair cells. For hair cell quantification, we imaged the entire cochlea using a ×40 lens on the Zeiss microscope and used ImageJ software to quantify the myosin 7a-positive cells. The average number of hair cells per 200 *μ*m in the middle turn of the cochlea was calculated from each group. Exact numbers of cochlear explants (*n*) are indicated in the legends.

### 2.15. Statistical Analysis

Statistical analyses were performed using GraphPad Prism statistical software (version 6, GraphPad Software, Inc., San Diego, CA). Comparison between two groups was analyzed by unpaired Student's *t*-test and comparison between multiple groups by one-way ANOVA. A *p* value < 0.05 was considered statistically significant for all tests.

## 3. Results

### 3.1. Ferroptosis Can Be Induced in HEI-OC1 Cells

**Figure 1:**
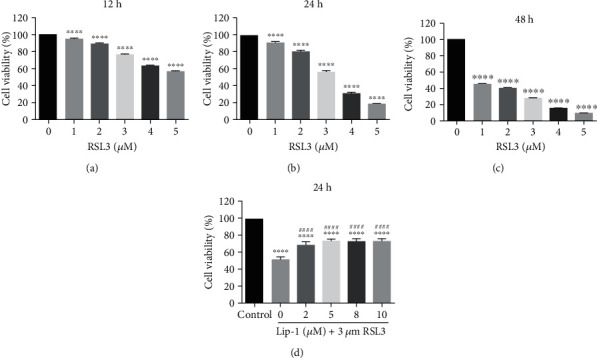
Ferroptosis exists in HEI-OC1 cells. (a–c) Cell viability was assessed after exposure to different concentrations of RSL3 for different times (12 h, 24 h, and 48 h). (d) Cell viability was assessed after exposure to 3 *μ*M RSL3 and different concentrations of Lip-1. Cell viability was measured by CCK8 kit. Values were represented as the mean ± s.e.m.^∗∗∗^*p* < 0.001 and ^∗∗∗∗^*p* < 0.0001 vs. the controls; ^####^*p* < 0.0001 vs. the group treated with RSL3 alone.

To test whether ferroptosis is induced in HEI-OC1 cells, twenty-four hours after the start of the HEI-OC1 cell culture, we replaced the culture medium with fresh medium with RSL3, a ferroptosis inducer, at increasing concentrations for 12 h, 24 h, and 48 h (Figures 1(a)–1(c)). CCK8 assay revealed that treatment with RSL3 at a concentration greater than 3 *μ*M for 24 h significantly reduced the cell viability to ~50% compared with the nontreated controls; thus, a RSL3 concentration of 3 *μ*M was used for the subsequent experiments. To determine if inhibitor of ferroptosis could protect HEI-OC1 cells from RSL3-induced cell death, cells were cotreated with Lip-1 concentrations of 0, 2, 5, 8, and 10 *μ*M for 24 h. We observed a significant protective effect of Lip-1 above 5 *μ*M, compared with culture treated with RSL3 alone (Figure 1(d)).

### 3.2. Lip-1 Protects Viability of HEI-OC1 Cells against Neomycin Damage

**Figure 2:**
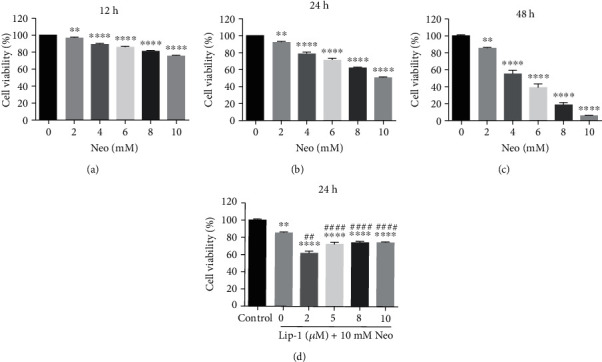
Effects of Lip-1 on neomycin-induced ototoxicity in HEI-OC1 cells. (a–c) HEI-OC1 cells were treated with or without various concentrations of neomycin for different times (12 h, 24 h, and 48 h). (d) Neomycin-damaged cells were cotreated with or without Lip-1. Cell viability was measured by CCK8 kit. Values were represented as the mean ± s.e.m.^∗∗^*p* < 0.01 and ^∗∗∗∗^*p* < 0.0001 vs. the control group; ^##^*p* < 0.01 and ^####^*p* < 0.0001 vs. the neomycin group.

HEI-OC1 cells were first exposed to increasing concentrations of neomycin (0, 2, 4, 6, 8, or 10 mM) [17, 22] for three different times (12 h, 24 h, and 48 h) to ascertain an optimal *in vitro* neomycin ototoxicity model. As shown in Figure 2, neomycin significantly decreased the cell viability in a dose- and time-dependent manner (Figures 2(a)–2(c)). Based on the cell viability data, we selected 10 mM neomycin treatment for 24 h as an appropriate condition for HEI-OC1 cell injury to the study of neomycin ototoxicity, as the viability was significantly decreased to ~50% compared with the nontreated control group. In order to test if Lip-1 was able to protect HEI-OC1 cells from neomycin-induced ototoxicity, the cells were cotreated with Lip-1 and neomycin. Using CCK8 assay, we confirmed that cell viability in the presence of Lip-1 was indeed significantly higher than in the absence of Lip-1 (Figure 2(d)). Based on these data, we selected 5 *μ*M Lip-1 as the optimal concentration for subsequent experiments. Next, to explore whether the protection mechanisms of Lip-1 are partly due to apoptosis, we measured the apoptotic rate of HEI-OC1 cells by flow cytometry. The results showed that the proportions of dead and apoptotic cells markedly increased in the neomycin group compared to the control group. Lip-1 remarkably decreased the cell death, while it had no significant effect on reducing the apoptotic cells (Supplemental Fig. 1A-C). In addition, Lip-1 reduced cell death induced by neomycin measured by TUNEL labeling (Supplemental Fig. 1D and E).

**Figure 3:**
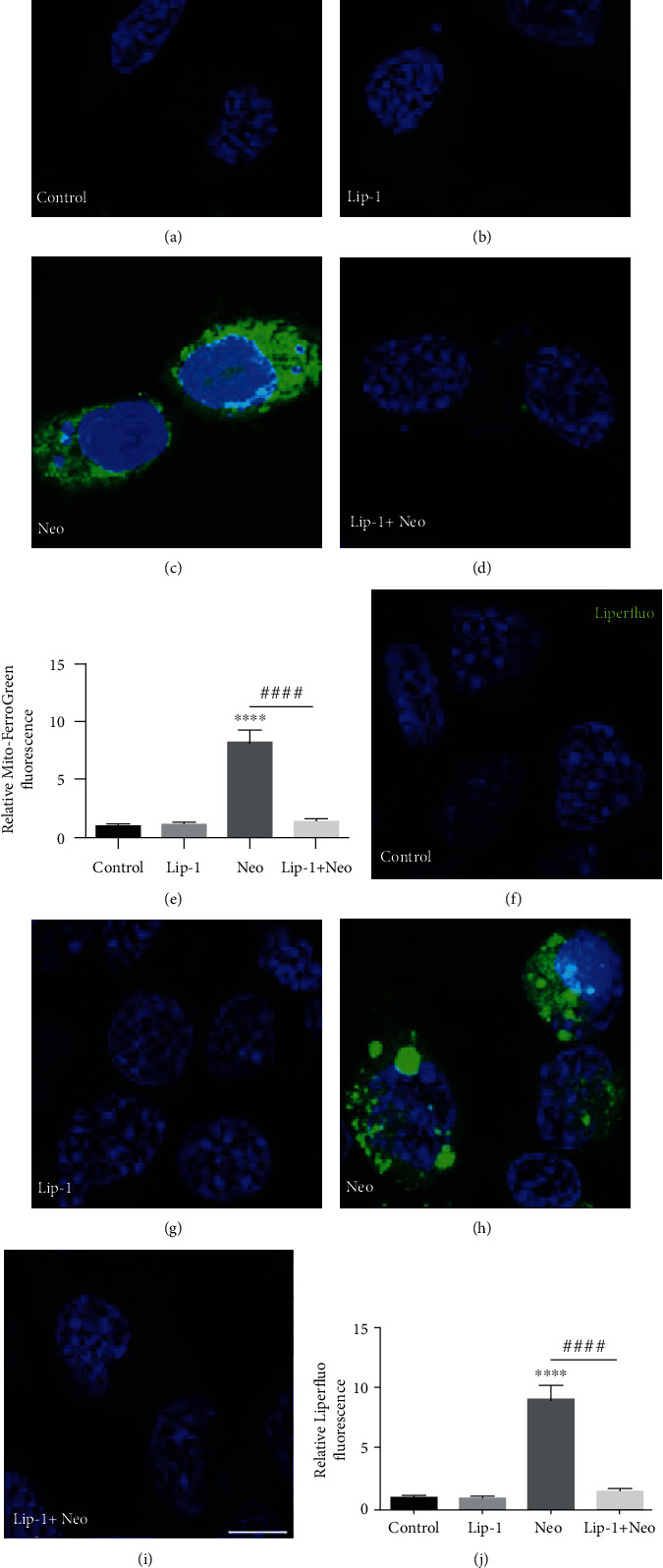
Fluorescence microscopic analysis for detecting mitochondrial Fe^2+^ and lipid peroxidation in neomycin-damaged HEI-OC1 cells. Representative images of Mito-FerroGreen staining in the (a) control, (b) Lip-1, (c) neomycin (Neo), and (d) Lip-1+neomycin cotreatment (Lip-1+Neo); scale bars indicate 10 *μ*m. (e) Quantification of Mito-FerroGreen staining in HEI-OC1 cells confirmed a significant reduction with Lip-1 administration. Representative images of Liperfluo staining in the (f) control, (g) Lip-1, (h) neomycin (Neo), and (i) Lip-1+neomycin cotreatment (Lip-1+Neo). Scale bars indicate 10 *μ*m. (j) Quantification of Liperfluo staining in HEI-OC1 cells confirmed a significant reduction with Lip-1 administration. Values were represented as the mean ± s.e.m.^∗∗∗∗^*p* < 0.0001 vs. the control group; ^####^*p* < 0.0001 vs. the neomycin group, *n* = 6.

Mito-FerroGreen, a novel mitochondria-targeted fluorescent probe for the detection of ferrous ion (Fe^2+^) in live cell mitochondria, and Liperfluo, a fluorescent probe for a detection of lipid peroxides, were used to monitor ferroptosis. As shown in Figure 3, neomycin exposure induced an increase in intracellular Fe^2+^ and lipid peroxidation which could be significantly reduced by Lip-1 cotreatment (Figure 3). These findings suggested that inhibition of ferroptosis was able to ameliorate neomycin-induced damage to HEI-OC1 cells.

### 3.3. Lip-1 Alleviates Neomycin-Induced ROS Generation

**Figure 4:**
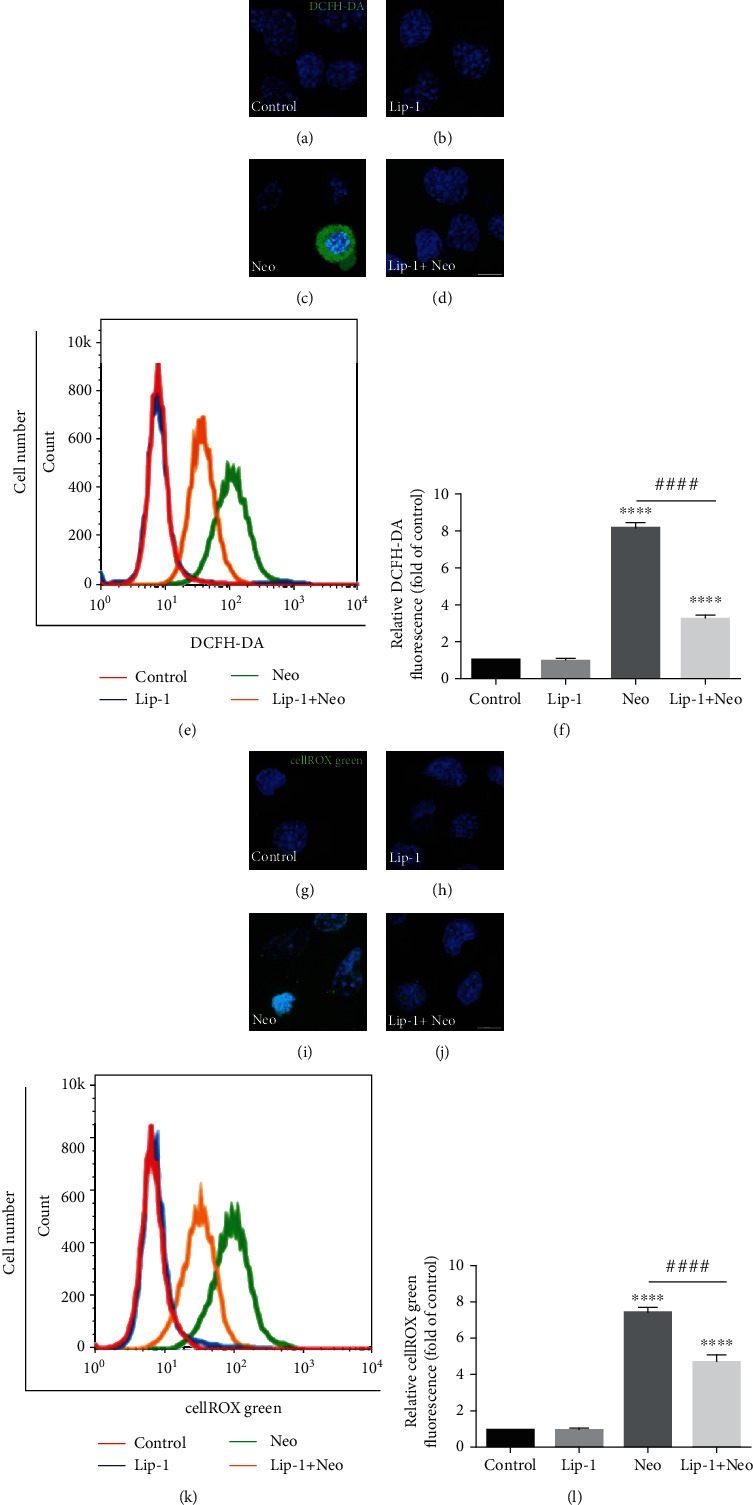
Effects of Lip-1 on ROS production in neomycin-damaged HEI-OC1 cells. Representative images of DCFH-DA staining in the (a) control, (b) Lip-1, (c) neomycin (Neo), and (d) Lip-1+neomycin cotreatment (Lip-1+Neo). Scale bars indicate 10 *μ*m. (e) Flow cytometry data confirmed the results in (a)–(d). (f) Quantification of the flow cytometry data. Representative images of cellROX green staining in the (g) control, (h) Lip-1, (i) neomycin (Neo), and (j) Lip-1+neomycin cotreatment (Lip-1+Neo). Scale bars indicate 10 *μ*m. (k) Flow cytometry data confirmed the results in (g)–(j). (l) Quantification of the flow cytometry data. Values were represented as the mean ± s.e.m.^∗∗∗∗^*p* < 0.0001 vs. the control group; ^####^*p* < 0.0001 vs. the neomycin group.

To confirm whether Lip-1 is associated with ROS generation, ROS production was measured by immunofluorescence and flow cytometric analysis with DCFH-DA staining in HEI-OC1 cells. We found that DCFH-DA intensity was significantly increased in the neomycin treatment group compared with controls, indicating that neomycin damage induced overproduction of ROS (Figures 4(a) and 4(c)). In contrast, DCFH-DA intensity was significantly reduced in the Lip-1 cotreatment group compared with the neomycin only group (Figures 4(d)–4(f)). In addition, production of ROS can also be examined by using the ROS indicator dye cellROX green, labeling a series of intracellular compartments. Immunofluorescence and flow cytometry results demonstrated that cellROX signal was significantly increased in the neomycin treatment group compared with the undamaged controls (Figures 4(g) and 4(i)), and cotreatment of neomycin and Lip-1 significantly attenuated cellROX fluorescence in HEI-OC1 cells compared with neomycin alone (Figures 4(j)–4(l)). Neomycin also increased ROS accumulation in a time-dependent manner; in contrast, Lip-1 treatment significantly inhibited neomycin-induced ROS generation in HEI-OC1 cells (Supplemental Fig. 2). These results suggested that Lip-1 significantly alleviated neomycin-induced overproduction of ROS in HEI-OC1 cells, which likely contribute to the observations of improved cell viability.

### 3.4. Lip-1 Mitigates Neomycin-Induced Mitochondrial Dysfunction

**Figure 5:**
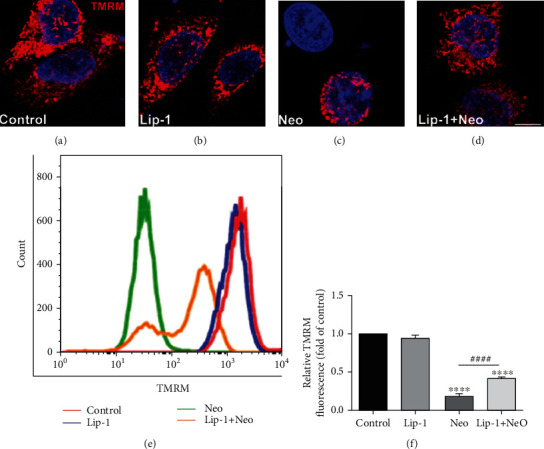
Effects of Lip-1 on mitochondrial membrane potential in neomycin-damaged HEI-OC1 cells. Representative images of TMRM staining in the (a) control, (b) Lip-1, (c) neomycin (Neo), and (d) Lip-1+neomycin cotreatment (Lip-1+Neo). Scale bars indicate 10 *μ*m. (e) Flow cytometry data confirmed the results in (a)–(d). (f) Quantification of the flow cytometry data. Values were represented as the mean ± s.e.m.^∗∗∗∗^*p* < 0.0001 vs. the control group; ^####^*p* < 0.0001 vs. the neomycin group.

Next, in order to assess whether Lip-1 provides protection against neomycin treatment by mitigating mitochondrial dysfunction, we measured the mitochondrial membrane potential (ΔΨ*m*) by TMRM fluorescence staining assay. Compared with untreated controls, TMRM fluorescence intensity was significantly decreased following neomycin exposure (Figures 5(a) and 5(c)). In contrast, Lip-1 cotreatment largely prevented neomycin-induced mitochondrial dysfunction (Figures 5(d)–5(f)). No significant difference in TMRM intensity was observed in cells treated with Lip-1 alone (Figure 5(b)). Collectively, these results demonstrated that Lip-1 could mitigate neomycin exposure-induced mitochondrial dysfunction.

### 3.5. Lip-1 Preserves Mitochondrial Morphology in Neomycin-Treated HEI-OC1 Cells

Furthermore, mitochondrion-specific dye MitoTracker Green was used to specifically label the mitochondria of HEI-OC1 cells (Supplemental Fig. 3). Our results showed that the filiform mitochondria were decreased while the punctate mitochondria were increased in neomycin-treated cells, which was markedly restored by Lip-1 (Supplemental Fig. 3). These results demonstrated that Lip-1 is able to preserve mitochondrial morphology.

### 3.6. Lip-1 Protects Mouse Cochlear Hair Cells against Neomycin-Induced Damage

**Figure 6:**
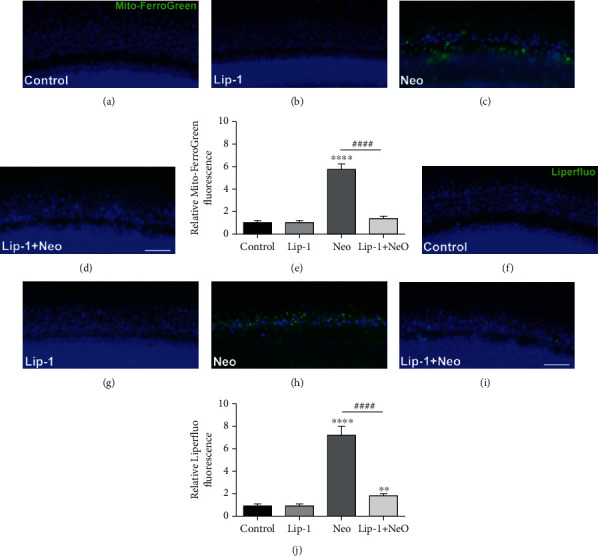
Fluorescence microscopic analysis for detecting mitochondrial Fe^2+^ and lipid peroxidation in neomycin-damaged cochlear hair cells. Representative images of Mito-FerroGreen staining in the (a) control, (b) Lip-1, (c) neomycin (Neo), and (d) Lip-1+neomycin cotreatment (Lip-1+Neo). Scale bars indicate 50 *μ*m. (e) Quantification of Mito-FerroGreen staining in cochlear hair cells confirmed a significant reduction with Lip-1 administration. Representative images of Liperfluo staining in the (f) control, (g) Lip-1, (h) neomycin (Neo), and (i) Lip-1+neomycin cotreatment (Lip-1+Neo). Scale bars indicate 50 *μ*m. (j) Quantification of Liperfluo staining in HEI-OC1 cells confirmed a significant reduction with Lip-1 administration. Values were represented as the mean ± s.e.m.^∗∗^*p* < 0.01 and ^∗∗∗∗^*p* < 0.0001 vs. the control group; ^####^*p* < 0.0001 vs. the neomycin group, *n* = 10.

**Figure 7:**
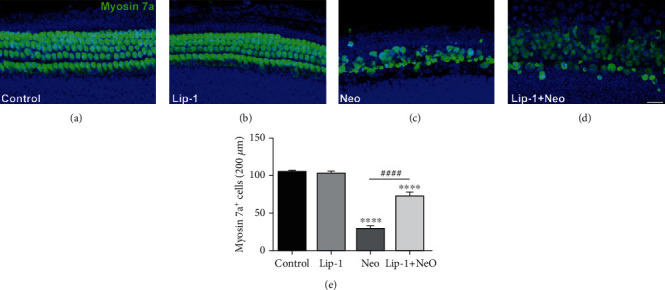
Effects of Lip-1 on neomycin-induced cochlear hair cell loss *in vitro*. Representative images of hair cell staining labeled with myosin 7a in the (a) control, (b) Lip-1, (c) neomycin (Neo), and (d) Lip-1+neomycin cotreatment (Lip-1+Neo). Scale bars indicate 20 *μ*m. (e) Quantification of myosin 7a-positive hair cells in the middle turns of different groups. Values were represented as the mean ± s.e.m.^∗∗∗∗^*p* < 0.0001 vs. the control group; ^####^*p* < 0.0001 vs. the neomycin group, *n* = 6.

In order to assess whether ferroptosis contributes to neomycin exposure-induced hair cell damage, cochlear explant treated with 1 mM neomycin for 6 h was stained with Mito-FerroGreen and Liperfluo probes. As shown in Figure 6, compared to undamaged controls, Mito-FerroGreen and Liperfluo signals were significantly increased after treatment with neomycin, and these increases were significantly inhibited by Lip-1 cotreatment (Figure 6). We next investigated whether Lip-1 protects hair cells against neomycin-induced damage in cultured cochlear explants from postnatal day (P) 2 mice. Hair cells with normal nuclei and labeled with myosin 7a, a specific hair cell marker [23, 24], were considered to be surviving hair cells and were counted. Our results showed that cochlea explants treated with 1 mM neomycin for 6 h displayed extensive degeneration of HCs in the cochlea (Figures 7(a) and 7(c)). In contrast, cotreatment with Lip-1 and neomycin exhibited protective effects against the neomycin-induced loss of HCs (Figures 7(d) and 7(e)). Cochlear explants treated with Lip-1 alone did not exhibit any damage to the HCs (Figure 7(b)). To investigate the mechanism of the protective effect of Lip-1 in neomycin-treated cochlear explants, we examined the expression of GPX4 [25]. The results showed that neomycin significantly decreased the expression of GPX4 compared with the control group, measured by western blot assay. Lip-1 treatment significantly reduced the deficit (Supplemental Fig. 4).

**Figure 8:**
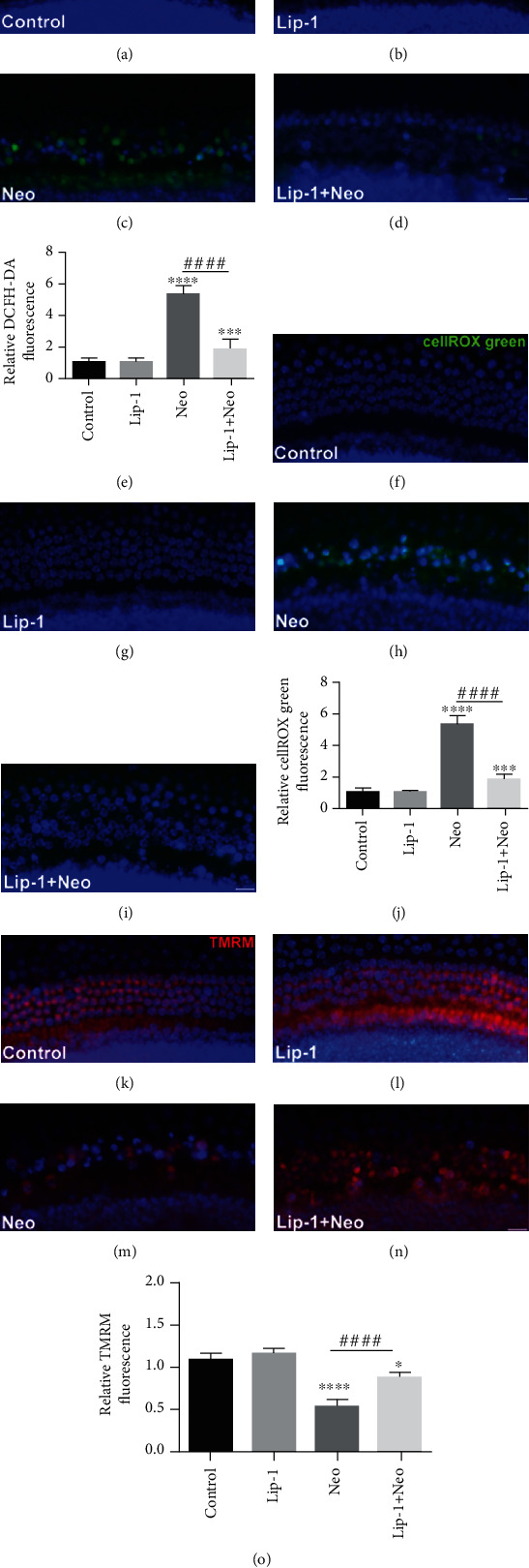
Effects of Lip-1 on ROS production and mitochondrial membrane potential in neomycin-damaged cochlear hair cells. Representative images of DCFH-DA staining in the (a) control, (b) Lip-1, (c) neomycin (Neo), and (d) Lip-1+neomycin cotreatment (Lip-1+Neo). (e) Quantification of DCFH-DA staining in cochlear hair cells. Representative images of cellROX green staining in the (f) control, (g) Lip-1, (h) neomycin (Neo), and (i) Lip-1+neomycin cotreatment (Lip-1+Neo). (j) Quantification of cellROX green staining in cochlear hair cells. Representative images of TMRM staining in the (k) control, (l) Lip-1, (m) neomycin (Neo), and (n) Lip-1+neomycin cotreatment (Lip-1+Neo). (o) Quantification of TMRM staining in cochlear hair cells. Scale bars indicate 20 *μ*m. Values were represented as the mean ± s.e.m.^∗^*p* < 0.05, ^∗∗^*p* < 0.01, ^∗∗∗^*p* < 0.001, and ^∗∗∗∗^*p* < 0.0001 vs. the control group; ^###^*p* < 0.001 and ^####^*p* < 0.0001 vs. the neomycin group, *n* = 10.

DCFH-DA and cellROX were used to investigate the effects of Lip-1 treatment on intracellular ROS levels in cochlear hair cells after neomycin treatment. The immunohistochemistry results showed that the ROS levels were increased after neomycin exposure compared to the undamaged controls (Figures 8(a)–8(j)), and Lip-1 treatment significantly decreased the ROS levels in cochlear hair cells compared with the neomycin alone (Figures 8(a)–8(j)). To further confirm whether the iron dysregulation is associated with ROS production and hair cell death induced by neomycin, we used cellROX staining to investigate the effect of the ferroptosis inducer RSL3 on the ROS production in cochlear hair cells after neomycin treatment with or without ROS scavenger N-acetylcysteine (NAC). Immunofluorescence results showed that RSL3 treatment markedly enhanced ROS production in neomycin-damaged cochlear hair cells. Nonetheless, treatment with ROS inhibitor NAC significantly inhibited RSL3-induced ROS generation in neomycin-damaged cells (Supplemental Fig. 5). We next used TMRM staining to investigate the effects of Lip-1 treatment on the mitochondrial function of cochlear hair cells after neomycin exposure. Our immunohistochemistry results showed that when treated with Lip-1, the TMRM intensity was significantly increased in neomycin-damaged cochlear hair cells (Figures 8(k)–8(o)).

**Figure 9:**
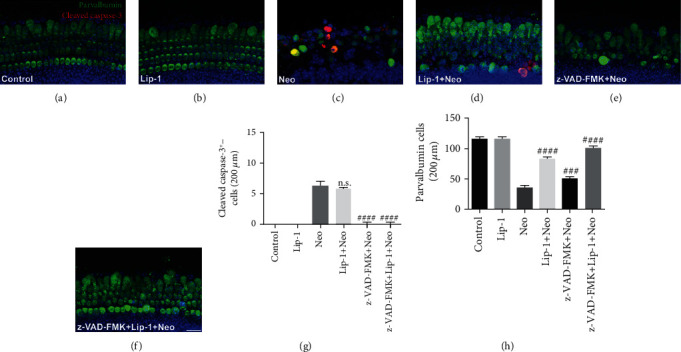
Effects of Lip-1 on apoptosis in neomycin-damaged cochlear hair cell. Representative images of cleaved caspase-3 and parvalbumin staining in the middle turns of the cochleae from (a) control, (b) Lip-1, (c) neomycin (Neo), (d) Lip-1+neomycin cotreatment (Lip-1+Neo), (e) z-VAD-FMK+neomycin (z-VAD-FMK+Neo), and (f) z-VAD-FMK+Lip-1+neomycin cotreatment (z-VAD-FMK+Lip-1+Neo). Scale bars indicate 20 *μ*m. (g) Quantification of cleaved caspase-3-positive cells in the middle turns of different groups. (h) Quantification of parvalbumin-positive hair cells in the middle turns of different groups. Values were represented as the mean ± s.e.m.^###^*p* < 0.001 and ^####^*p* < 0.0001 and n.s. no significant vs. the neomycin group, *n* = 10.

To investigate whether the decreased apoptosis contributes to the decreased sensitivity of cochlear hair cells to neomycin damage after Lip-1 treatment, we treated the cochlear hair cells with Lip-1 in the absence or presence of z-VAD-FMK (a pan-caspase inhibitor). The expression of apoptotic protein cleaved caspase-3 in cochlear hair cells was upregulated when detected with immunocytochemistry following the challenge of neomycin, and this effect was markedly reversed by the pretreatment with 50 *μ*M z-VAD-FMK. In contrast, Lip-1 at a dose of 5 *μ*M had no significant effects on that of cleaved caspases-3 (Figure 9). Of note, although the numbers of parvalbumin, a specific hair cell marker [26, 27], positive hair cells in the pretreated z-VAD-FMK group were increased after neomycin treatment compared to the cochlear explants exposed to neomycin without z-VAD-FMK pretreatment, more hair cells were observed in cochlear explants pretreated with Lip-1 than in cochlear explants pretreated with z-VAD-FMK. Collectively, these findings suggest that ferroptosis and apoptosis contribute to cell death induced by neomycin in cochlear hair cells, but Lip-1-mediated hair cell protection was caspase independent, at least in our experimental conditions.

## 4. Discussion

Aminoglycosides are widely used in the treatment of severe infections; however, ototoxicity is a serious side effect among patients [28]. Reducing this aminoglycoside-induced ototoxic effect is an important problem for hearing studies. The present study provides evidence that the ferroptosis inhibition might serve as a new target to reduce aminoglycoside-induced ototoxicity.

Ferroptosis is a type of newly discovered iron-dependent programmed cell death that is characterized by the intracellular overproduction of ROS and lipid peroxidation, but independent from caspase-mediated cell death [9]. In recent years, accumulating studies have reported ferroptosis occurring in multiple pathological situations, such as neurodegenerative diseases [13]. Previous studies have proposed a novel mechanism of aminoglycoside ototoxicity which was based on observations of iron chelation and free radical generation [29–31], suggesting that aminoglycoside gentamicin acted as an iron chelator and that the formation of an iron-aminoglycoside complex was a preliminary step in free radical generation and subsequent hair cell death. Furthermore, Song and Schacht [32] have demonstrated that iron chelators are more efficient than various radical scavengers in attenuating gentamicin-induced hearing loss *in vivo* in the guinea pig. The fact that the iron chelator deferoxamine effectively attenuated another aminoglycoside neomycin-induced hearing loss further confirmed that iron availability is indeed a critical factor in aminoglycoside ototoxicity [33]. Studies from this mechanism led to promising therapeutic prevention of aminoglycoside-induced ototoxicity by the use of iron chelators and radical scavengers. So far, the role of ferroptosis in aminoglycoside-induced ototoxicity is unknown. Because the ROS accumulation is associated with ototoxicity pathophysiology, we speculated that ferroptosis may be correlated with aminoglycoside-induced ototoxicity.

In this study, we found that RSL3 treatment induced an increase in intracellular Fe^2+^ and lipid peroxidation which could be significantly reduced by treatment with liproxstatin-1 (Lip-1), the first *in vivo* efficacious ferroptosis inhibitor, as assessed using the Mito-FerroGreen and Liperfluo probes. Hence, these data strongly indicated that ferroptosis could be induced in HEI-OC1 cells. We next analyzed the correlation between ferroptosis and neomycin. We found that hair cell apoptosis was not the only type of neomycin-induced damage. Further experiments showed that ferroptosis occurred in neomycin-damaged HEI-OC1 cell line and cochlear hair cells. Treatment with Lip-1 could rescue ferroptosis induced by neomycin in hair cells, suggesting that ferroptosis inhibitor could become a novel therapeutic strategy for preventing hearing loss caused by aminoglycoside ototoxicity.

It has been demonstrated that mitochondria served a major role in ototoxic drug-induced ototoxicity [21, 34]. For instance, the previous study showed that inhibition of PRMT6 using pharmacological and genetic interventions significantly prevents hair cells from aminoglycoside- and cisplatin-induced damage by decreasing ROS generation and results in preservation of mitochondrial function, supporting the notion that targeting mitochondria might improve protection against ototoxicity-induced hearing loss [21]. In the present study, it was observed that neomycin promoted cell death, ROS production, and iron accumulation in HEI-OC1 cells, which was consistent with those previously reported [21, 34]. Pretreatment with Lip-1 significantly decreased neomycin-induced cell death and ROS production and increased mitochondrial membrane potential in HEI-OC1 cells, implying that ferroptosis represented a central mechanism of aminoglycoside-induced ototoxicity and ferroptosis inhibitor could preserve mitochondrial function, thereby mitigating hair cell death secondary to neomycin exposure.

The common ferroptotic mechanisms result from system X_c_^−^ inhibition, GSH depletion, GPX4 inactivation, lipid peroxidation, and iron dysregulation. GPX4 is a lipid repair enzyme, which reduces phospholipid hydroperoxides within membranes and lipoproteins and inhibits the initiation of ferroptosis [11, 35]. Inactivation of GPX4 activity or degradation of GPX4 induces rapid accumulation of lipid peroxides and contributes to ferroptosis, and knocking out GPX4 is embryonically lethal [10, 36, 37]. GPX4 has also been shown to protect cells from apoptosis [38, 39] and necroptosis [40], suggesting that it plays a protective role in cell death. Reduced GSH, a basal cofactor of GPXs, plays a role in the antioxidant defense, and the GSH/GPX4 axis is critical for the regulation of redox [10]. GSH depletion reduces the activity of GPX4, which is a key event upstream of mitochondrial dysfunction, leading to ferroptosis [10, 41]. Further research is required to analyze the spatial and temporal distribution patterns of these potential ferroptosis regulators by using appropriate pharmacologic and genetic inhibitory approaches in neomycin-triggered hair cell injury, such as GPX4, GSH, and xCT.

The more complete picture of molecular mechanisms underlying neomycin-induced ferroptosis in hair cell needs further exploration. Previous studies have reported that the mitogen-activated protein kinase (MAPK) and AKT pathway has been implicated in erastin-induced ferroptosis in cancer cells [41, 42] and oxidative stress-induced hair cell damage [43, 44]. Erastin could activate the JNK and p38 MAPK signaling pathway and trigger ferroptosis, and inhibition of JNK and p38 MAPK activation significantly decreased the erastin-induced cytotoxicity, further confirming the role of MAPK in erastin-induced ferroptosis in HL-60 cells [41, 45]. MAPK signaling is also associated with OGD/R-induced ferroptosis in Sertoli cells [46]. Inactivation of p38 MAPK was demonstrated to prevent OGD/R-induced cell ferroptosis confirming the role of p38 MAPK in ferroptosis. However, whether these signaling pathways serve roles in neomycin-induced ferroptosis in hair cells remains an open question. Additionally, although ferroptosis contributes to neomycin ototoxicity in hair cells, other cell death pathways cannot be ruled out in these assays. Future research could focus on how ferroptosis interacts with other cell deaths, such as apoptosis, necrosis, and autophagy, at the molecular level and how these pathways could be integrated in hair cells.

## 5. Conclusions

In summary, our findings revealed that Lip-1 protected hair cells from aminoglycoside-induced ototoxic damage by inhibition of ferroptosis in a cultured HEI-OC1 cell line and in a neonatal mouse cochlear explant model. Our findings therefore suggest that ferroptosis inhibition might have clinical applications for the attenuation of aminoglycoside-induced hearing loss. Further studies extending these findings to *in vivo* mouse models utilizing multiple ototoxic agents are required to advance ferroptosis inhibitor as a potential otoprotectant in patients.

## Data Availability

The data used to support the findings of this study are available from the corresponding authors upon request.

## Supplementary Materials

Supplementary Materials Supplemental Figure 1: Effects of lip-1 on neomycin-induced apoptosis inHEI-OCI cells. Supplemental Figure 2: Lip-1 reduced the neomycin-induced ROS production in HEI-OCI cells in a time-dependent manner. Supplemental Figure 3: Effects of LIP-1 on mitochondrial morphology in neomycin-damaged HEI-OC1 cells. Supplemental Figure 4: Effects of LIP-1 on GPX4 expression in neomycin -damaged cochlear explants. Supplemental Figure 5: Effects of RSL3 on the ROS production in cochlear hair cells after neomycin treatment with or without NAC.
