# Erratum to “Liproxstatin-1 Protects Hair Cell-Like HEI-OC1 Cells and Cochlear Hair Cells against Neomycin Ototoxicity”

**DOI:** 10.1155/2021/9873282

**Published:** 2021-12-13

**Authors:** Zhiwei Zheng, Dongmei Tang, Liping Zhao, Wen Li, Jinghong Han, Bing Hu, Guohui Nie, Yingzi He

**Affiliations:** ^1^ENT Institute and Department of Otorhinolaryngology, Eye & ENT Hospital, Fudan University, Shanghai 200031, China; ^2^NHC Key Laboratory of Hearing Medicine (Fudan University), Shanghai 200031, China; ^3^Department of Otolaryngology and Institute of Translational Medicine, Shenzhen Second People's Hospital/The First Affiliated Hospital of Shenzhen University Health Science Center, Shenzhen 518035, China

In the article titled “Liproxstatin-1 Protects Hair Cell-Like HEI-OC1 Cells and Cochlear Hair Cells against Neomycin Ototoxicity” [1], the incorrect figure files were introduced by the Editorial staff during the publication process. The figures should be corrected as follows:

## Figures and Tables

**Figure 1:**
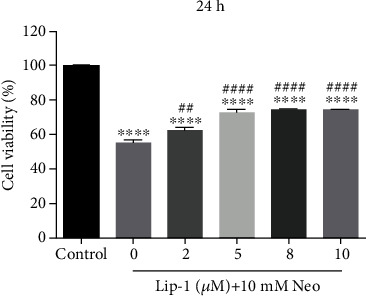
(d) Neomycin damaged cells were cotreated with or without Lip-1. Cell viability was measured by CCK8 kit. Values were represented as the mean ± s.e.m. ^∗∗^*p* < 0.01 and ^∗∗∗∗^*p* < 0.0001 vs. the control group; ^##^*p* < 0.01 and ^####^*p* < 0.0001 vs. the neomycin group.

**Figure 2:**
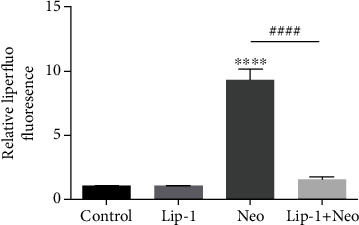
(j) Quantification of Liperfluo staining in HEI-OC1 cells confirmed a significant reduction with Lip-1 administration. Values were represented as the mean ± s.e.m. ^∗∗∗∗^*p* < 0.0001 vs. the control group; ^####^*p* < 0.0001 vs. the neomycin group, *n* = 6.

**Figure 3:**
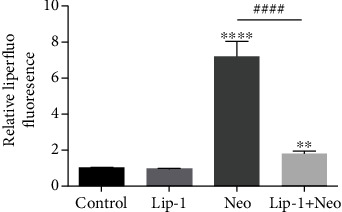
(j) Quantification of Liperfluo staining in cochlear hair cells confirmed a significant reduction with Lip-1 administration. Values were represented as the mean ± s.e.m. ^∗∗^*p* < 0.01 and ^∗∗∗∗^*p* < 0.0001 vs. the control group; ^####^*p* < 0.0001 vs. the neomycin group, *n* = 10.

**Figure 4:**
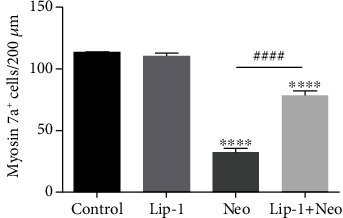
(e) Quantification of myosin 7a-positive hair cells in the middle turns of different groups. Values were represented as the mean ± s.e.m. ^∗∗∗∗^*p* < 0.0001 vs. the control group; ^####^*p* < 0.0001 vs. the neomycin group, *n* = 6.

**Figure 5:**
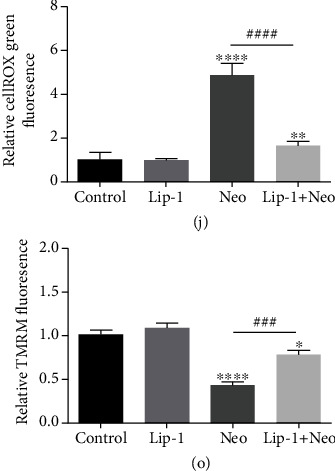
(j) Quantification of cellROX green staining in cochlear hair cells. (o) Quantification of TMRM staining in cochlear hair cells. Scale bars indicate 20 *μ*m. Values were represented as the mean ± s.e.m. ^∗^*p* < 0.05, ^∗∗^*p* < 0.01, ^∗∗∗^*p* < 0.001, and ^∗∗∗∗^*p* < 0.0001 vs. the control group; ^###^*p* < 0.001 and ^####^*p* < 0.0001 vs. the neomycin group, *n* = 10.
