# Supplementary material for: Liproxstatin-1 Protects Hair Cell-Like HEI-OC1 Cells and Cochlear Hair Cells against Neomycin Ototoxicity
Source: Oxid Med Cell Longev. 2020 Dec 1;2020:1782659. doi: 10.1155/2020/1782659 (PMC7725559; doi:10.1155/2020/1782659)
Supplement: Supplementary Materials — Supplemental Figure 1: Effects of lip-1 on neomycin-induced apoptosis inHEI-OCI cells. Supplemental Figure 2: Lip-1 reduced the neomycin-induced ROS production in HEI-OCI cells in a time-dependent manner. Supplemental Figure 3: Effects of LIP-1 on mitochondrial morphology in neomycin-damaged HEI-OC1 cells. Supplemental Figure 4: Effects of LIP-1 on GPX4 expression in neomycin -damaged cochlear explants. Supplemental Figure 5: Effects of RSL3 on the ROS production in cochlear hair cells after neomycin treatment with or without NAC. [file 1782659.f1.docx]

**Liproxstatin-1 protects hair cell-like HEI-OC1 cells and cochlear hair cells against neomycin ototoxicity**

Zhiwei Zheng^a,b^*, Dongmei Tang^a,b^*, Liping Zhao^a,b^*, Wen Li^a,b^, Jinghong Han^c^, Bing Hu^c^, Guohui Nie^c#^, Yingzi He^a,b#^

a ENT institute and Department of Otorhinolaryngology, Eye & ENT Hospital, Fudan University, Shanghai, 200031, China

b NHC Key Laboratory of Hearing Medicine (Fudan University), Shanghai, 200031, China

c Department of Otolaryngology and Institute of Translational Medicine, Shenzhen Second People’s Hospital/ the First Affiliated Hospital of Shenzhen University Health Science Center, Shenzhen, 518035, China.

* Zhiwei Zheng, Dongmei Tang and Liping Zhao contributed equally to this work.

# Correspondence should be addressed to:

Guohui Nie, Shenzhen Second People's Hospital, 3002 Sungang W Rd, Futian Qu, Shenzhen Shi, Guangdong Sheng, 518029, China; E-mail: [nieguohui@email.szu.edu.cn](mailto:nieguohui@email.szu.edu.cn)

Yingzi He, ENT institute and Department of Otorhinolaryngology, Eye & ENT Hospital, Fudan University, 83 Fenyang Road, Shanghai, 200031, China; Tel: +86-21-64377134; E-mail: yingzihe09611@126.com

**Supplemental Materials**

**
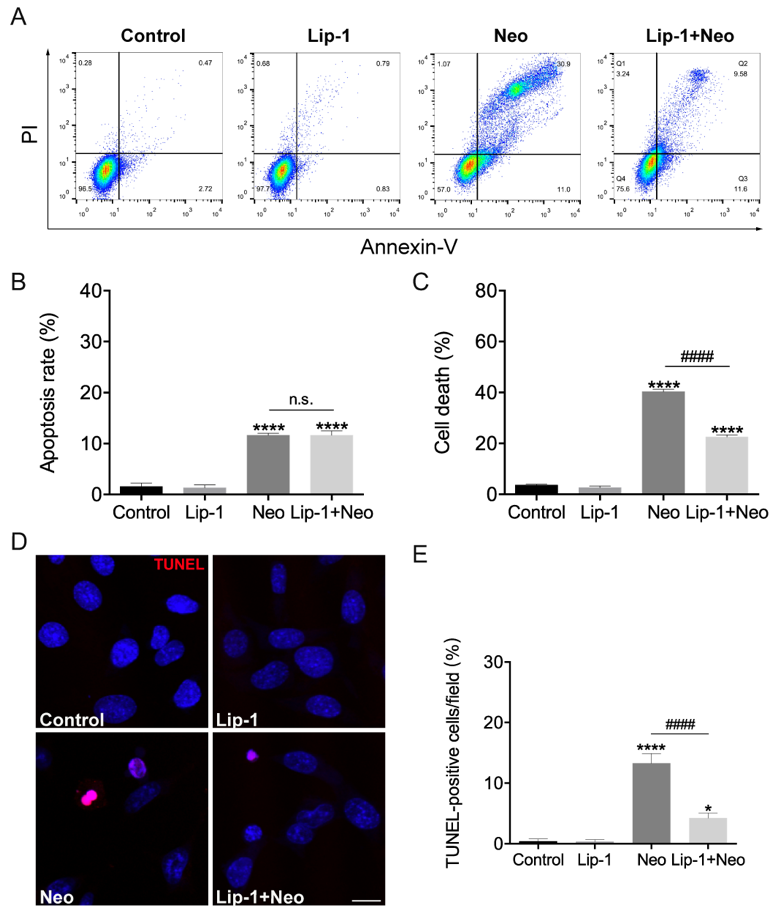
**

**Supplemental Fig. 1.**  Effects of Lip-1 on neomycin-induced apoptosis in HEI-OC1 cells. (A-C) Cell apoptosis was determined by flow cytometry using Annexin V and PI staining kit. (D-E) Representative images of TUNEL staining from different treatments (D) and quantification of the data (E) were shown. Scale bars = 20 μm. Data were presented as the mean ± s.e.m. of n = 6 comparable sections from a representative experiment performed three times. **p* < 0.05 and *****p* < 0.0001 vs. the control group; ^####^*p* < 0.0001 n.s. no significant. vs neomycin group.

**
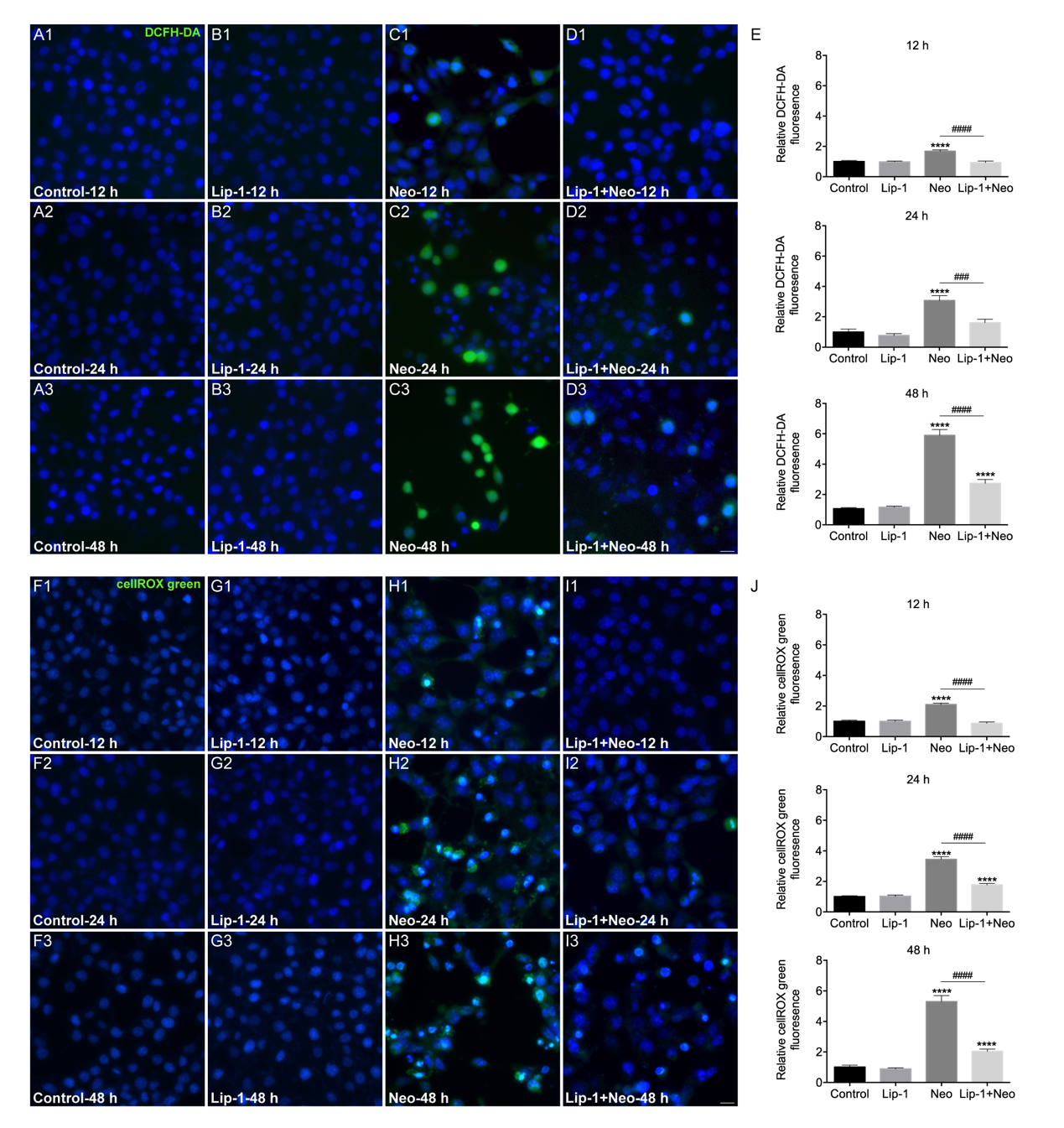
**

**Supplemental Fig. 2.** Lip-1 reduced the neomycin-induced ROS production in HEI-OC1 cells in a time-dependent manner. (A-D) Immunofluorescence analysis of the DCFH-DA staining in control (A1-A3), Lip-1 (B1-B3), neomycin (Neo; C1-C3), and Lip-1 + neomycin cotreatment (Lip-1+Neo; D1-D3) at 12 h, 24 h, and 48 h. (E) Quantification of DCFH-DA staining in HEI-OC1 cells. (F-I) Immunofluorescence analysis of the cellROX green staining in control (F1-F3), Lip-1 (G1-G3), neomycin (Neo; H1-H3), and Lip-1 + neomycin cotreatment (Lip-1+Neo; I1-I3) at 12 h, 24 h, and 48 h. (J) Quantification of cellROX green staining in HEI-OC1 cells**.** Scale bars indicate 20 μm. Values were represented as the mean ± s.e.m. *****p* < 0.0001 vs. the control group; ^####^*p* < 0.0001 vs. the neomycin group, n = 8-12.


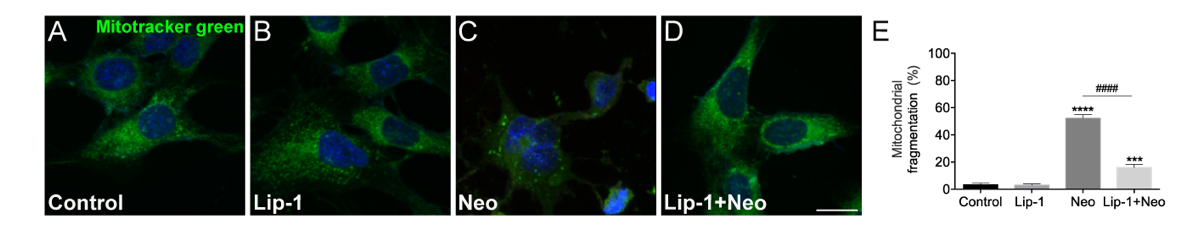


**Supplemental Fig. 3.** Effects of Lip-1 on mitochondrial morphology in neomycin-damaged HEI-OC1 cells. (A-D) Representative images of Mitotracker green staining in the control (A), Lip-1 (B), neomycin (Neo; C), and Lip-1 + neomycin cotreatment (Lip-1+Neo; D). Green fluoresence indicates mitochondria, while blue fluoresence indicates nuclear. Scale bars indicate 20 μm. (E) Quantification of the fragmented mitochondria. Values were represented as the mean ± s.e.m. ****p* < 0.001 and *****p* < 0.0001 vs. the control group; ^####^*p* < 0.0001 vs. the neomycin group, n = 8.


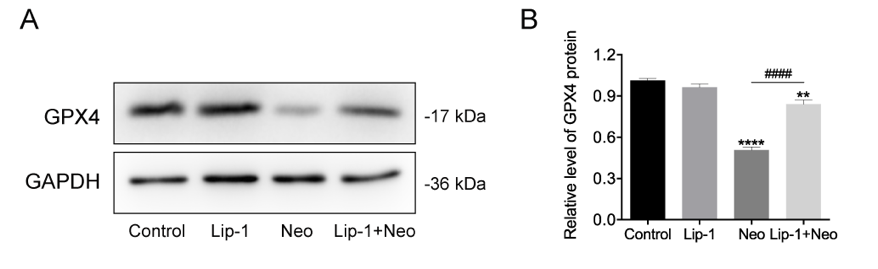


**Supplemental Fig. 4.** Effects of Lip-1 on GPX4 expression in neomycin-damaged cochlear explants. (A-B) Western blot analysis of GPX4. Quantification of expression of GPX4 protein assessed by immunoblot. The data is shown as mean ± s.e.m. of three independent experiments. ***p* < 0.01, *****p* < 0.0001 vs. the control; ^####^*p* < 0.0001 vs. the neomycin group.


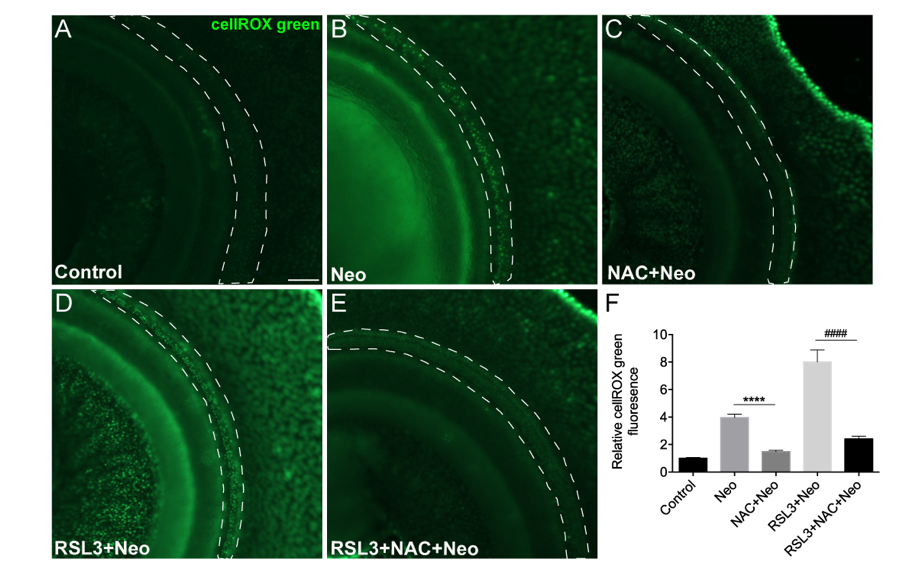


**Supplemental Fig. 5.** Effects of RSL3 on the ROS production in cochlear hair cells after neomycin treatment with or without NAC. (A-E) Representative images of cellROX green staining in the control (A), neomycin (Neo; B), NAC + neomycin cotreatment (NAC+Neo; C), RSL3 + neomycin cotreatment (RSL3+Neo; D), and RSL3 + NAC + neomycin cotreatment (RSL3+NAC+Neo; E). Scale bars indicate 100 μm. (F) Quantification of cellROX green staining. Values were represented as the mean ± s.e.m. *****p* < 0.0001 vs. the neomycin group; ^####^*p* < 0.0001 vs. the RSL3 + neomycin cotreatment group, n = 10.
